# Aging, Alzheimer's, and *APOE* genotype influence the expression and neuronal distribution patterns of microtubule motor protein dynactin-P50

**DOI:** 10.3389/fncel.2015.00103

**Published:** 2015-03-25

**Authors:** Orwa Aboud, Paul A. Parcon, K. Mark DeWall, Ling Liu, Robert E. Mrak, W. Sue T. Griffin

**Affiliations:** ^1^Donald W. Reynolds Department of Geriatrics, University of Arkansas for Medical SciencesLittle Rock, AR, USA; ^2^Department of Biology, Brigham Young UniversityIdaho, Rexburg, ID, USA; ^3^Department of Pathology, University of Toledo Health Sciences CampusToledo, OH, USA; ^4^Geriatric Research, Education, Clinical Center, Central Arkansas HealthCare SystemLittle Rock, AR, USA

**Keywords:** aging, Alzheimer, *APOE* genotype, βAPP, dynactin-P50, motor proteins, P-tau, synaptophysin

## Abstract

Reports from neural cell cultures and experimental animal studies provide evidence of age- and disease-related changes in retrograde transport of spent or misfolded proteins destined for degradation or recycling. However, few studies address these issues in human brain from those who either age without dementia and overt neuropathology, or succumb to Alzheimer's; especially as such propensity may be influenced by *APOE* genotype. We studied the expression and distribution of the dynein subunit dynactin-P50, the β amyloid precursor protein (βAPP), and hyperphosphorylated tau (P-tau) in tissues and tissue sections of brains from non-demented, neuropathology-free patients and from Alzheimer patients, with either *APOE* ε3,3 or *APOE* ε4,4. We found that advanced age in patients without dementia or neuropathological change was associated with coordinated increases in dynactin-P50 and βAPP in neurons in pyramidal layers of the hippocampus. In contrast, in Alzheimer's, βAPP and dynactin were significantly reduced. Furthermore, the dynactin-P50 and βAPP that was present was located primarily in dystrophic neurites in Aβ plaques. Tissues from Alzheimer patients with *APOE* ε3,3 had less P-tau, more βAPP, dynactin-P50, and synaptophysin than did tissues from Alzheimer patients carrying *APOE* ε4,4. It is logical to conclude, then, that as neurons age successfully, there is coordination between retrograde delivery and maintenance and repair, as well as between retrograde delivery and degradation and/or recycling of spent proteins. The buildup of proteins slated for repair, synaptic viability, transport, and re-cycling in neuron soma and dystrophic neurites suggest a loss of this coordination in Alzheimer neurons. Inheritance of *APOE* ε3,3 rather than *APOE* ε4,4, is associated with neuronal resilience, suggestive of better repair capabilities, more synapses, more efficient transport, and less hyperphosphorylation of tau. We conclude that even in disease the ε3 allele is neuroprotective.

## Introduction

Fast-axonal transport is an essential part of normal neuronal function (Paschal and Vallee, 1987; Brady, 1991), and transport failures manifest as a dying-back of axons from the synapse to the neuronal soma, a phenomena that occurs prior to the neuronal loss characteristic of Alzheimer's disease (AD) (Kanaan et al., 2013). The retrograde transport complex dynein is a large multi-subunit complex, which attaches to its cargo by the dynactin-P50 subunit (Chen et al., 2014). Dynein-mediated transport is speculated to be mostly regulated by the dynactin complex (Stokin and Goldstein, 2006). The active dynein complex is important in alignment of microtubules and is, at least partially, responsible for the growth of microtubules into the growth cone of axons (Ahmad et al., 2006). These functions suggest that dynein is needed for neuronal survival, especially for the survival of those neurons with long axons that function to connect CNS neuronal somas with distant targets in the periphery (Ebneth et al., 1998; Heerssen et al., 2004). Further, dynein-mediated axonal transport depends on interactions between neurotrophic factors, their receptors, and the dynein complex. For example, decreased brain-derived neurotropic factor (BDNF) is associated with decreased retrograde transport (Heerssen et al., 2004).

Among the endosomes transported by dynein, many contain Aβ or βAPP; the latter being expressed in response to neuronal stress imposed by neural injury (Rosen et al., 1989; Kimura et al., 2009). Elevated expression of βAPP is a neuronal response believed to be neuroprotective (Masliah et al., 1997) based on findings in mice deficient in βAPP; such mice display deficits in long term potentiation and memory (Dawson et al., 1999). Normal transport of excess βAPP and Aβ to the soma for degradation is necessary to terminate reactions, which over time may facilitate Aβ deposition. For example, as a result of breakdown in endosome transport, Aβ vesicles in the cell build up, causing further neuronal stress, further increases in βAPP production, and inhibition of Aβ reuptake and processing, leading to Aβ deposition (Kimura et al., 2009). Such deposition has been noted in neurons adjacent to Aβ plaques, and is characterized by a failure to mount appropriate neuronal acute phase responses such as elevation of βAPP (Barger et al., 2008).

The genesis and stability of microtubules is dependent on appropriately phosphorylated tau, a principal microtubule-associated protein that is necessary for genesis and stability of the microtubule tracks that are used in intracellular transport (Yoon et al., 2008) and in maintenance of the unipolarity of the axon, which is important for dynein and kinesin motor functions (Ebneth et al., 1998). Tau is normally distributed along the axon in a decreasing gradient, with low tau concentration near the cell body, and higher concentrations near the axonal ending, for appropriate attachment of motor proteins (Dixit et al., 2008). In the absence of regulation, there is neuritic beading, axonal swelling, and bulbous neurites, which is presumably due to stearic hindrance or microtubule dysfunction in the areas of the swelling (Tan et al., 2007; Kimura et al., 2009). With either broken tracks or an obstacle in its path, tau begins to build up in the soma, which may result from, or may lead to, its hyperphosphorylation and ultimately formation of neurofibrillary tangles (NFTs) in cell bodies and apical dendrites, in distal dendrites as neuropil threads, as well as in the enlarged dystrophic neurites in Aβ plaques (Gotz et al., 2006).

Several studies dating back to as early as 1967 suggest that microtubule-dependent transport is impaired in Alzheimer's disease (Suzuki and Terry, 1967) (for review see Stokin and Goldstein, 2006). The correlation between neurofibrillary tangles and dementia severity (Gotz et al., 2006) may be related to abnormal phosphorylation of tau and resultant changes in axonal transport of hyperphosphorylated tau and βAPP, as well as other cellular entities (Greenberg and Kosik, 1995). For instance, the presence in axons and neurites of abnormal swellings containing neurofibrillary tangles, phagocytic elements, and mitochondria (Pilling et al., 2006; Tan et al., 2007) suggests that motor protein dysfunction and/or microtubule loss (Cash et al., 2003) contribute to these anomalies. This is of considerable interest as failures in endosomal functions that are dependent on retrograde transport by the dynein system leave the neuron unable to rid itself of unwanted or misfolded proteins, and spent organelles. It is also important to note that in both Alzheimer's and Down's syndrome, endosomal changes are among the earliest pathological anomalies (Cataldo et al., 1996, 2003), suggesting that failures in this system are particularly powerful as they occur early and are persistent, with middle and end stage accumulation of unwanted proteins in synaptic areas.

The role of *APOE* genotype in risk for development of Alzheimer's is well known (Saunders et al., 1993; Strittmatter et al., 1993; LaDu et al., 1994; Weisgraber and Mahley, 1996), and the potential additive effect of having *APOE* ε4 allele(s) on the risk for developing Alzheimer's in risk-conferring neurological conditions such as in head trauma (Nicoll et al., 1995; Reinvang et al., 2013) and in temporal lobe epilepsy (Mackenzie and Miller, 1994; Gouras et al., 1997; Aboud et al., 2012, 2013) has been reported. *APOE* genotype has also been shown to impact age-associated cognitive status (Vemuri et al., 2010, 2014) and is associated with vocabulary loss with age (Baxter et al., 2003), especially in the context of more limited education and less intellectual stimulation (Bunce et al., 2014). Only recently has it been suggested that inheritance of specific *APOE* genotypes may be more related to the ability of one genotype to confer neuronal resilience rather than neurodegeneration; that is, allelic combinations without ε4 are more resilient than combinations with ε4 (Caesar and Gandy, 2012).

To assess whether age and/or Alzheimer's and *APOE* genotype are related to changes in neuronal and tissue levels of the neuronal acute phase response protein βAPP, the retrograde microtubule motor protein dynactin-P50, accumulation of hyperphosphorylated tau in NFTs, or synaptic loss (as denoted by a reduction in the synaptic marker synaptophysin), human brain tissue samples and tissue sections from brains of cognitively intact patients at different ages (age-matched control, or AMC), and from Alzheimer patients were immunoreacted with antibodies that specifically recognize dynactin-P50, βAPP, synaptophysin, and hyperphosphorylation sites on the tau in neurofibrillary tangles. Dynactin-P50 expression in neurons in both hippocampal dentate gyrus and pyramidal neuron areas increased with normal aging, while such expression in Alzheimer patients was markedly less. Similarly, the expression of βAPP was elevated with aging, but not with Alzheimer's. Disease-associated decreases in neuronal βAPP and dynactin-P50 expression were accompanied by an accumulation of βAPP, dynactin-P50, and neurofibrillary tangles in neuron somas and dystrophic neuritic compartments, especially in neuritic plaques. The relative levels of βAPP, dynactin-P50, and synaptophysin were higher in tissue from *APOE* ε3,3 carriers than in *APOE* ε4,4 carriers, while the levels of hyperphosphorylated tau were higher in *APOE* ε4,4 than *APOE* ε3,3 carriers.

## Materials and methods

### Patients and specimens

Hippocampal tissues from 23 brains obtained at autopsy from 11 neurologically normal individuals (AMC, ages 16–81 years, all *APOE* ε3,3) and 12 Alzheimer patients (ages 68–90 years; 4 *APOE* ε3,4 and 8 *APOE* ε3,3) were used for immunohistochemical analysis of cellular expression of dynactin-P50, βAPP, and hyperphosphorylated tau. To assess the potential role of *APOE* ε3,3 vs. *APOE* ε4,4 in dynactin-P50 and dynein/cargo interactions, hippocampal tissue from 11 Alzheimer patients (5 *APOE* ε3,3 and 6 *APOE* ε4,4) and 4 *APOE ε3,3* neurologically normal controls was evaluated with respect to the relative tissue levels of dynactin-p50, hyperphosphorylated tau, and synaptophysin. All tissues were neuropathologically evaluated and the diagnoses of AD or AMC were made according to National Institute of Aging-Reagan guidelines^1^.

### Fluorescent immunohistochemistry

As described in a previous study (Barger et al., 2008), paraffin was removed in xylene from 7 μm paraffin-embedded tissue sections and rehydrated in graduated ethanol solutions to deionized water. Sections were incubated in 3% H_2_O_2_in 97% methanol for 30 min at room temperature and washed 3 times with PBS. For βAPP, dynactin P-50, and AT8 labeling, tissue sections were pretreated by boiling in 1 mmol/L of EDTA (pH 8.0) for 10 min at medium power in a microwave oven. After 3 rinses in PBS, all sections were blocked in 80% Dulbecco's Modified Eagle's Medium [DMEM], 19% fetal calf serum, 1% bovine serum albumin for 1 h at room temperature. After PBS rinses, sections were incubated with primary antibody solutions overnight at room temperature: mouse anti-dynactin P-50 (1:1000) (BD Bioscience, San Jose, CA), mouse anti-βAPP (1:100) (Zymed, Grand Island, NY), and mouse anti-hyperphosphorylated tau (AT8) (1:2000) (Thermo Scientific, Pittsburg, PA). After PBS rinses, a pre-diluted horse anti-mouse immunoglobulin G conjugated to peroxidase (Vector, Burlingame, CA) was further diluted 1:5 for detection of dynactin P-50, hyperphosphorylated tau (AT8), and βAPP primary antibodies. Development of the immunofluorescent signal was achieved by signal amplification with Alexa Fluor 350, 488, and 594 tyramide (Molecular Probes, Eugene, OR), using the manufacturer's recommended conditions. Before the addition of the second or third primary antibody in double and triple labeling experiments, slides were rinsed in PBS, incubated in 0.01 N HCl for 10 min at room temperature to inactivate peroxidase, rinsed again in PBS, and subjected to antigen-specific pretreatments as necessary. Previous to secondary antibody application, lipofuscin staining was blocked by pretreatment with Sudan Black B dissolved in 80% ethanol (Sigma, St. Louis, MO). Tissue sections were counterstained with 100 ng/ml 4′,6 diamino-2-phenylindole dihydrochloride (DAPI, Invitrogen, Grand Island, NY) for 5 min at room temperature to visualize nuclei. To prevent fading of immunofluorescence, the tissue sections were mounted with Vectashield mounting medium (Vector, Burlingame, CA).

**Figure 1:**
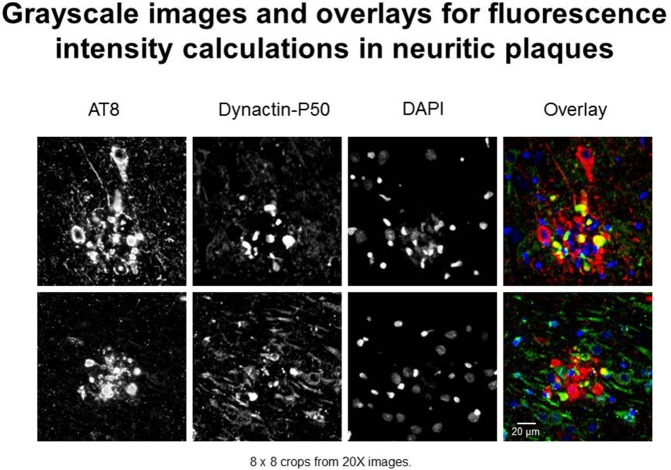
**Examples of immunofluorescent images used in neuritic plaque color overlays and for grayscale quantification**. Separate channel images were taken and overlaid, and colocalization was assessed based on color combinations, e.g., Red + Green = Yellow. Red represents hyperphosphorylated tau (AT8) and green represents dynactin-P50 (DynP50).

In this study, we use “expression” to mean fluorescence intensity. The fluorescence intensity of βAPP, dynactin, and p-tau in immunoreacted tissues were examined according to standardized laboratory practices using a Nikon Eclipse E600 microscope with a Y-FL epifluorescence attachment (Barger et al., 2008; Aboud et al., 2012, 2013). A CoolSNAP ES digital camera (Roper Scientific, Ottobrunn, Germany) was used to capture images from the hippocampus, and the parahippocampal gyrus at 20× or 40× magnification under identical conditions (exposure times). Thresholding and total fluorescence intensity calculations were derived from gray-scale images (Figure 1)—captured using NIS-Elements BR3 (Nikon.com) and MetaVue 6.2r2 software (Molecular Devices, Sunnyvale, CA) at a minimum threshold range of 300 and 600 for dynactin P-50 and βAPP—in neurons, i.e., cells with nuclear diameters greater than 8 μm and in neurites, i.e., plaque-associated, non-nucleated immunoreactive entities (Figure 1).

In brief, quantification protocols were as follows: initial image samples were taken for each patient to determine optimal exposure times, such that all signal was clearly detected and no pixels were saturated. Once exposure time was determined, images from all slides were taken at the same exposure time, with multiple images taken in areas of interest in each sample. NIS-Elements software denotes pixel saturation, and fields containing such saturation due to blood vessels or other artifacts were excluded from data collection and calculation. Intensity calculations are averaged for each individual, with the average intensity considered as a single data point for statistical analysis.

### Duolink proximity ligation assay

Duolink PLA kits were purchased from Sigma, and were used according to manufacturer's protocols. In brief, slides were treated similar to immunofluorescent protocols above, with the replacement of fluorescent antibodies with oligonucleotide-conjugated secondary antibodies (termed PLUS and MINUS) that are complementary and form a circular nucleotide sequence if the two secondary antibodies are within 40 nm distance from each other. Slides are treated with a ligase following the secondary antibodies, and then a fluorescent signal is produced by rolling circle elongation from the circular DNA strand formed from the PLUS and MINUS antibodies. Duolink was performed with the dynactin-P50 and AT8 primary antibodies listed above.

### Western immunoblot analysis

Proteins were extracted from tissue with a lysis buffer comprised of 50 mM Tris-HCl (pH 7.5), 150 mM NaCl, 1% Nonidet P40, 0.5% sodium deoxycholate and 0.1% SDS; lysates were quantified using a Micro BCA assay reagent kit (Pierce, Rockford IL) as described previously (Li et al., 1998). Aliquots (100 μg each) were loaded onto 10% SDS-polyacrylamide gels, subjected to electrophoresis at 70V for 20 min and 90V for 1.5 h, and transferred to nitrocellulose membranes. Membrane blots were blocked in I-Block Buffer (Applied Biosystem Inc., Bedford, MA) for 45 min, then incubated overnight separately at 4°C with primary antibodies to: P-tau (AT8, 1:1000); dynactin-P50 (1:1000); synaptophysin 1:1000; or actin (1:5000), incubated for 1 h at room temperature with alkaline phosphatase-conjugated secondary antibody, and developed using the Western-Light™ Chemiluminescent Detection System (Applied Biosystem Inc., Bedford MA). Autoradiographs were digitized and analyzed using NIH ImageJ software, version 1.60. Results are reported as steady-state levels, relative to actin.

### Statistical analysis

Group differences were determined by Wilcoxon-Mann-Whitney Rank Sum Test for significance, with two tails and α = 0.05. Results are expressed as mean ± SEM.

## Results

### βAPP and dynactin-P50 expression increased with age

**Figure 2:**
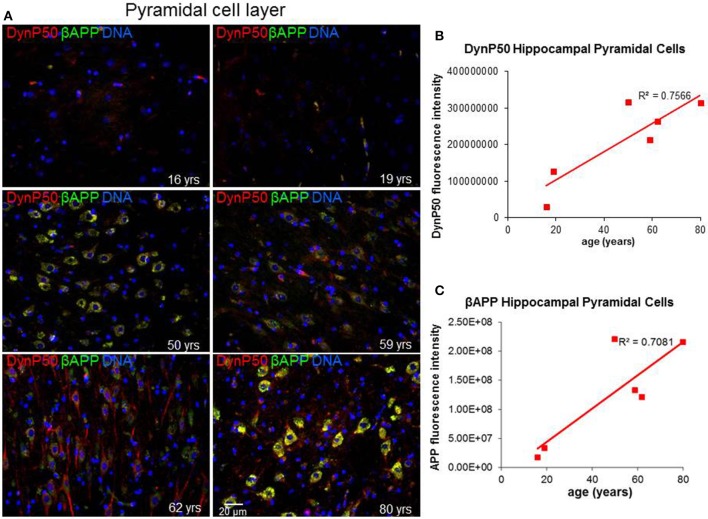
**Age-related changes in expression of dynactinP-50 (DynP50) and β-amyloid precursor protein (βAPP)**. Dynactin-P50 and βAPP were detected by immunofluorescence in tissue sections from hippocampus at the level of the lateral geniculate nucleus from 6 neurologically normal individuals across an age span of 16–80 years. **(A)** Representative immunofluorescent images from the six individuals; blue represents DAPI staining of cellular DNA, red represents dynactinP-50, green represents βAPP, and yellow represents colocalization of dynactinP-50 and βAPP. Images were digitized at 20× magnification. Scale bar = 20 μm. Quantitation of dynactinP-50 **(B)** and βAPP **(C)** immunofluorescence intensity was obtained by thresholding gray-scale images and integrating pixels as described in Materials and Methods section. Values reflect the mean of 6 images per specimen.

Due to the elongated morphology of neurons and their unique functions, precise and timely delivery of specific proteins and organelles by motor proteins must be tightly maintained. In particular, retrograde transport of spent proteins or damaged organelles is very important and is predicted to change with age, as oxidative and inflammatory stresses accumulate with the wear and tear of time. To investigate this prediction, we ask a simple question: Are there changes in the expression of the regulatory cargo attachment protein dynactin-p50 and its cargo βAPP with increasing age in the absence of disease? Dynactin-P50 fluorescent intensity (expression) was noticeably elevated with increasing age, from teenage to the eighth decade, in pyramidal neurons in hippocampal tissue sections from neurologically and neuropathologically normal individuals (Figures 2A,B). This age-related increase in dynactin-P50 expression was accompanied by an increase in βAPP expression in neuronal somas and neuronal processes (Figures 2A,C), suggesting that in response to the normal wear and tear of time, βAPP and dynactin expression are increased for membrane repair and transport of cellular entities.

### βAPP and dynactin-P50 expression is diminished in Alzheimer hippocampus

**Figure 3:**
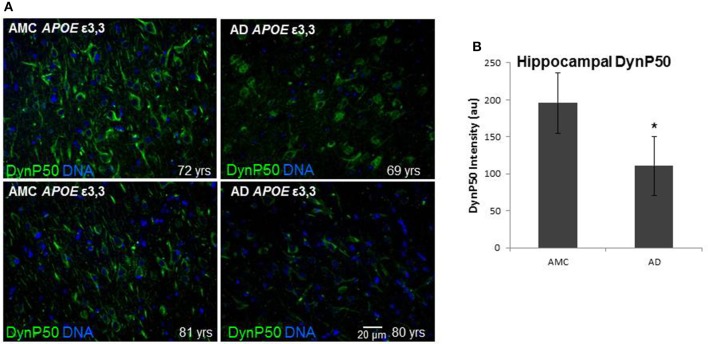
**Comparison of dynactin-P50 (DynP50) expression between neurologically normal controls (AMC) and Alzheimer (AD) patients. (A)** DynactinP-50 (green) in tissue sections from the hippocampal cell layer. Images were digitized at 20× magnification. Blue represents DAPI staining of cellular DNA. **(B)** Quantification of DynP50 in AD (*n* = 4) vs. neurologically normal control (AMC, *n* = 4). Values reflect the mean of 30 images of the pyramidal cell layer for AD and AMC. Significance determined by Wilcoxon-Mann-Whitney Rank Sum Test, with ^*^denoting *p* ≤ 0.05. Data reported as group mean with error bars denoting SEM.

**Figure 4:**
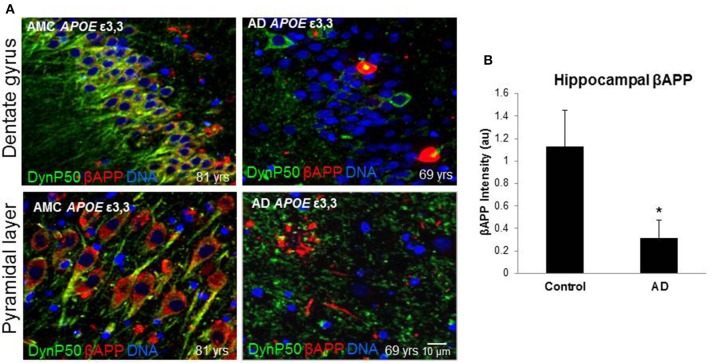
**βAPP levels are reduced in Alzheimer's (AD), and dynactin-P50 (DynP50) and βAPP localization is disrupted**. Dynactin-P50 (green) and βAPP (red) were detected by immunofluorescence in tissue sections from hippocampus. **(A)** Total APP intensity is decreased in AD cases. Blue represents DAPI staining of cellular DNA. Images were digitized at 40× magnification. Scale bar = 10 μm. **(B)** Overall βAPP fluorescence intensity is diminished in AD compared to neurologically normal controls (AMC). Significance determined by Wilcoxon-Mann-Whitney Rank Sum Test, with ^*^denoting *p* ≤ 0.05. Data reported as group mean with error bars denoting SEM.

In order to assess the effect of AD on the amount of βAPP and dynactin-p50 in the neurons of those with Alzheimer's disease, separate from the effect of normal aging, we selected cases with Alzheimer's disease, and compared them to control individuals that matched them closely in age (AMCs were within 1 year of their AD counterparts, with the total age range for both groups between 69 and 80). We found that there was a dramatic decrease in dynactin-P50 expression in analogous neurons in Alzheimer patients (Figures 3A,B). This decrease is suggestive of the idea that in AD, neurons are unable to transport proteins to the soma for degradation and recycling, perhaps accounting, at least in part, for the deleterious downstream consequences of a build-up of misfolded proteins and potentially-neurotoxic aggregates. The decrease in dynactin-P50 expression in neurons in tissue from Alzheimer patients, compared to that in tissue from age-matched neurologically normal individuals, was accompanied by a similarly marked decrease in neuronal βAPP (Figures 4A,B) and in βAPP mRNA, as we previously reported (Barger et al., 2008).

### Dynactin, βAPP, and neurofibrillary tangles are colocalized and distributed throughout neurons in Alzheimer's

**Figure 5:**
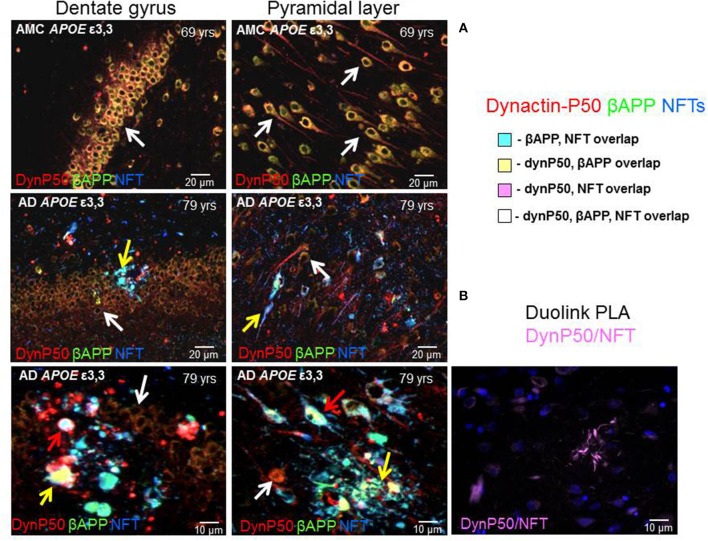
**Dynactin-P50 (DynP50), βAPP, and hyperphosphorylated tau in neurofibrillary tangles (NFT) localization in AD and neurologically normal controls (AMC). (A)** Dynactin-P50 (red), βAPP (green), and NFT (blue) were detected by immunofluorescence in hippocampal tissue of AD and neurologically normal individuals (AMC). In AMC (top row), note the absence of neurofibrillary tangles, and increased dynactin-P50 and βAPP compared to AD (middle and bottom rows). Further, DynP50 and βAPP appear brighter in somas of AMC vs. somas of AD without NFTs (white arrows). Dynactin-P50, APP, and NFT immunoreactivity was observed in anuclear bulbous neurites (yellow arrows). AD somas containing NFTs showed the highest immunoreactivity in AD for dynactin-P50, and βAPP, implying possible aggregation of all three antigens (red arrows). **(B)** Duolink proximity ligation assay (PLA) detects immunogens within 40 nm distance in tissue sections. Image shows neuritic plaque enriched with dynactin-P50 and P-tau in close proximity.

In neurologically normal individuals, the increased expression of dynactin-P50 and βAPP associated with advancing age was apparent in the soma and in the axon hillock of both dentate and pyramidal neurons (Figure 5, 1st row). In contrast, in Alzheimer tissue from analogous regions of hippocampus, the expression of dynactin-P50 and βAPP was dramatically reduced in dentate and pyramidal neurons (Figure 5, 2nd and 3rd row). Moreover, in dynactin-P50 and βAPP-immunoreactive neurons that also contained neurofibrillary tangles, there was an apparent preferential redistribution of all three antigens from neuronal somas to neuritic bulbous terminals (Figure 5, neuron soma, white arrows; anuclear neurites, yellow arrows). Large tangle-bearing pyramidal neurons were also noted to contain both βAPP and dynactin-P50. In fact, P-tau immunoreactive tangles, βAPP, and dynactin-P50 appear to be trapped in both soma and neurites (Figure 5, red arrows), which may be due, at least in part, to disruption of the entire (anterograde and retrograde) microtubule motor system, as might be inferred from previous studies demonstrating that endosomal transport and processing are disrupted in Down's syndrome and in Alzheimer's disease (Cataldo et al., 1996). To determine proximity of P-tau and dynactin-p50, we probed tissue with the Duolink Proximity Ligation Assay (PLA). This assay produces a fluorescent signal if two immunogens are within 40 nm distance by using complementary DNA strands on antibody probes with subsequent ligation and detection. In this way, we showed that dynactin-P50 and P-tau are indeed located within 40 nm of each other in neuritic plaques in AD (Figure 5B), strongly suggesting aggregation.

### Hyperphosphorylated tau is increased, and dynactin-P50 and synaptophysin are decreased in *APOE* ε4,4 Alzheimer patients compared to ε3,3

**Figure 6:**
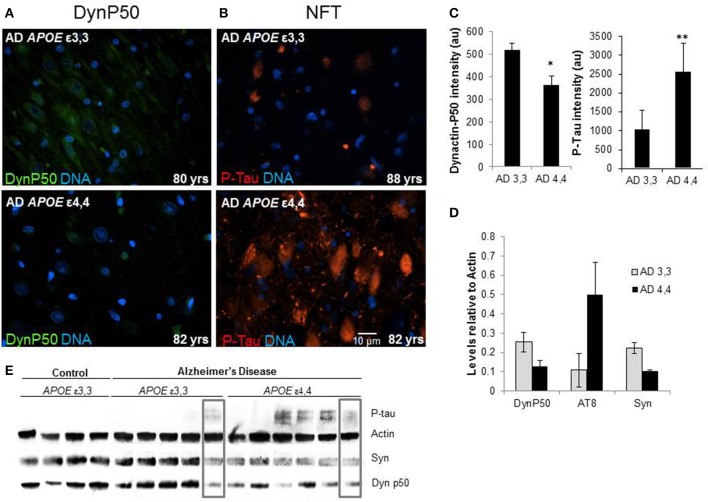
**Comparison of tissue levels of dynactin-P50 (DynP50), P-tau, and synaptophysin (Syn) in Alzheimer patients with *APOE* ε3,3 vs. ε4,4. (A)** Dynactin-P50 (green) was less in AD patients with *APOE* ε4,4, than in those with ε3,3, while **(B)** P-tau was increased in ε4,4. Representative images. **(C)** Fluorescence intensity quantification of P-tau and dynactin-P50 levels in AD patients with ε3,3 vs. ε4,4. **(D)** Steady state protein levels, relative to that of actin, of P-tau in Alzheimer patients with ε3,3 were lower than levels in those with ε4,4, while synaptophysin and dynactin-P50 levels were higher in ε3,3 compared with ε4,4. **(E)** Western blot comparison of protein levels in AD vs. Control, and between AD ε3,3 vs. AD ε4,4. Actin was used as loading control. Lanes in gray boxes demonstrate a patient with *APOE* ε3,3 genotype with similar levels of proteins to those in a patient with *APOE* ε4,4 genotype, suggestive of the possible presence of Alzheimer risk factors aside from *APOE* genotype. Wilcoxon-Mann-Whitney Rank Sum Test was used for statistical significance, with ^*^denoting *p* ≤ 0.05 and ^**^denoting *p* ≤ 0.01. Data reported as group mean with error bars denoting SEM.

Marked differences in fluorescence intensity were noted in AD patients depending on their *APOE* genotype. Those with ε3,3 genotypes had overall higher dynactin-P50 levels and lower P-Tau levels compared to their ε4,4 counterparts (Figures 6A,B, representative images, Figure 6C, fluorescence quantification). This was confirmed in Western blot: relative to actin steady-state levels, overall, the expression of hyperphosphorylated tau is higher in Alzheimer patients with *APOE* ε4,4 than in patients with *APOE* ε3,3, while dynactin and synaptophysin were lower (Figures 6D,E). As synaptophysin is commonly used as a marker for healthy functional synapses, this implies a connection between *APOE* genotype, retrograde transport, and synaptic integrity. Interestingly, in comparing the right-most lanes in the Alzheimer ε3,3 and ε4,4 western blot (Figure 6E, gray boxes), the hyperphosphorylated tau, synaptophysin, and dynactin-P50 levels are almost identical, perhaps explained by risk factors other than *APOE* genotype.

## Discussion

It is clear that successful brain aging, that is, aging free of dementia and of Alzheimer neuropathology at autopsy, is possible as many people live to old age without dementia (Evans et al., 1989; Paganini-Hill et al., 2015). This suggests that in successful aging, the brain meets the challenges of stresses that come with aging by bringing into play mechanisms that are necessary to maintain the status quo in a neuron. Such maintenance requires appropriate regulation (i.e., synthesis and activation) of proteins involved in the following: maintenance of membranes (e.g., βAPP); microtubule stability (e.g., tau); anterograde transport of proteins to where they are needed (via kinesin); retrograde transport of spent proteins and organelles (via dynein); and proper degradation and processing of spent proteins (ubiquitination for proteostasis). Numerous cell culture and experimental animal research studies have shown age-related changes in neuronal functions, many of which involve molecular mechanisms of neuronal transport systems. Our aim here was to define and characterize retrograde transport protein levels that accompany successful aging, and how those that aged successfully differed from those with Alzheimer's disease. We note here that in successful aging, there is a coordinated increase in dynactin and βAPP with advancing age. In contrast, many of the changes we discuss in successful aging may not occur in those with Alzheimer's disease, such that less βAPP and dynactin are produced in those with AD compared to their age-matched counterparts, and that the dynactin and APP which *is* produced is trapped in aggregates with hyperphosphorylated tau. Further, these deleterious effects are exacerbated by inheritance of *APOE* ε4,4 alleles in comparison to inheritance of *APOE* ε3,3. Our data is consistent with the idea that Alzheimer's disease constitutes a failure of neurons to sustain the levels of βAPP and dynactin-P50 necessary for the increased demands imposed by aging, especially in combination with other risk factors such as inheritance of *APOE* ε4.

### Normal aging

We focused on βAPP because it is the acute phase response protein in neurons (Barger et al., 2008) and is of particular importance in Alzheimer pathogenesis; and on dynein, in particular dynactin-P50, the cargo-binding protein of dynein, because βAPP is a prominent cargo for retrograde transport (Gunawardena et al., 2013). As we are interested in age-related effects, we restricted our cohort to patients without dementia and without neuropathology at autopsy. In our sample, we show that βAPP expression increases with age, and that this increase is accompanied by an increase in dynactin-P50. This is consistent with the idea that aging stress induces increased βAPP production, and that this increased βAPP necessitates a concomitant increase in transport proteins, as illustrated with dynactin-P50 in our study. Other studies have also pointed to differential expression of this system depending on age. Age-related increases in specific isoforms and fragments of βAPP (133 and 19 kDa, respectively) have been reported to occur with advancing age (Nordstedt et al., 1991), while decreases in microtubule density, the “tracks” upon which dynein complexes travel, are also occurring in an age-related manner (Cash et al., 2003). In 2007, Kimura et al. reported yet another effect, this time in aged monkeys: with increased age, the interaction between the dynein intermediate chain and dynactin was reduced, possibly due to phosphorylation state, and leading to decreased retrograde transport efficiency (Kimura et al., 2007). All of these effects may be responsible for possibly compensatory increases in dynactin we report here. We feel that it should be stressed here that individuals used in this study, even of advanced age, did not have disease, and the implication therein is that age alone is not sufficient to lead to neuropathology, as long as compensatory mechanisms function correctly.

### βAPP and dynactin in Alzheimer's disease

The story is different in individuals with neurodegenerative disease. Axonal transport disturbances are important factors in several neurological disorders, including Parkinson's disease (Chu et al., 2012) and amyotrophic lateral sclerosis (Ikenaka et al., 2012). Similar disturbances in axonal transport have been suggested to occur in Alzheimer's disease, based on the dystrophic appearance of axons and their bulbous neurites and dendrites in Alzheimer brain (Stokin and Goldstein, 2006). These characteristic defects in AD are suggestive of defects in axonal transport and have been widely reported on by many groups (Heerssen et al., 2004; Ahmad et al., 2006; Kimura et al., 2009). Our finding of dramatic reductions in neuronal dynein expression in Alzheimer brain, relative to that in neurologically normal controls of similar age (AMC), provides necessary support for the early suggestion by Terry (1998) that motor protein abnormalities are principal factors in synaptic pathology. Although this Alzheimer-related reduction in dynactin-P50 that we show relative to that in AMC is unlikely to be the only contributor to neuronal dysfunction, it is likely to be an important component of the endosomal-lysosomal system dysfunction that is related to defects in βAPP processing in AD (i.e., autophagy) (Cataldo et al., 1996), and in Down's (Cataldo et al., 2000). It may be the case that such transport protein dysfunction is secondary to primary lysosomal defects: Lee et al. demonstrated that lysosomal dysfunction was sufficient to cause axonal transport deficits of the like seen in Alzheimer's disease (Lee et al., 2011).

### Neurofibrillary tangles

The co-localization of dynein and βAPP as well as dynein and hyperphosphorylated tau in Alzheimer disease is consistent with the idea that—unlike the state of affairs in aged controls—spent or misfolded proteins, that would otherwise be transported back to the soma for degradation or recycling, are sequestered in aggregates in neurites and in somas. The presence of the retrograde motor protein dynactin-P50 in these aggregates, together with the fact that less dynactin-P50 is present overall in Alzheimer brain, supports a prominent role for retrograde transport dysfunction in Alzheimer pathogenesis. Moreover, the Alzheimer-related changes in dynein expression and distribution reported here, together with the sequestering of abnormal proteins in somas and terminals may contribute to the synaptic loss in AD that Terry and Katzman suggest is related to decreases in cognition and increased risk for precocious development of AD (Terry and Katzman, 2001).

The early finding of the importance of tau in the genesis and stabilization of microtubules (Black et al., 1986) has implications for our study in that the hyperphosphorylation of tau and subsequent formation of neurofibrillary tangles suggests sequestration of hyperphosphorylated tau in such tangles may decrease the amount of tau available for its normal functions to promote production of microtubule associated proteins for genesis and stability of microtubules (Trojanowski and Lee, 2005). This, along with the reported decrease in microtubule density with increasing age (Cash et al., 2003), the weakened synthesis of dynein, and the paucity of non-aggregated dynactin-P50 in Alzheimer neuron somas that we show here, suggest that in Alzheimer's, disruption in synthesis of transport proteins necessary for cellular and synaptic maintenance and repair leads to failures in proteostasis, synaptic loss, and neuronal dysfunction and death. While there is still no firmly established relationship between Aβ plaques and the presence and distribution of neurofibrillary tangles within neurons in Alzheimer's (Armstrong et al., 1993), our finding of both βAPP and dynactin-P50 in neurofibrillary tangle-laden bulbous neurites in plaques as well as in plaque-adjacent neuron somas suggests that plaques and plaque proximity may contribute to retrograde transport dysfunction and therefore to the buildup, rather than the degradation, of toxic aggregates such as hyperphosphorylated tau. Indeed, in Alzheimer's disease, increasing plaque complexity (diffuse to diffuse neuritic to dense-core neuritic) is associated with more βAPP-immunoreactive neurites and more activated astrocytes overexpressing S100B (Mrak et al., 1996), which could further exacerbate neuronal transport deficits, through its promotion of βAPP synthesis and neurite outgrowth and its dose-dependent induction of microtubule associated protein 2 (Li et al., 1998).

### *APOE* genotype

We present evidence here that *APOE* genotype has a part in the pathogenesis of these transport deficits. Our findings show that the tissue levels of dynactin-P50 and synaptophysin are lower in Alzheimer patients with *APOE ε* 4,4 compared to *APOE ε* 3,3, while hyperphosphorylated tau is elevated, suggesting that inheritance of ε3,3 confers a measure of neuronal resilience, even in the presence of Alzheimer's disease. Our findings regarding the influence of *APOE* genotype on the steady state levels of the motor protein dynactin-P50 and one of its potential cargoes, hyperphosphorylated tau, suggests that, compared to carriers of *APOE ε3,3*, carriers of *APOE* ε4,4 are at increased risk of excessive aggregation in neuronal soma and dystrophic neurites of spent and misfolded proteins. Therefore, these aggregated proteins are not available to proteosomes or lysosomes for degradation or re-cycling.

It is important to note that possession of *APOE ε3,3* genotype alone does not protect against disease-associated risk factors that themselves increase hyperphosphorylated tau. It does appear that as hyperphosphorylated tau increases above some level in carriers of *APOE ε3,3* with disease, there is *not* a corresponding increase in motor proteins. Moreover, *APOE ε3,3* carriers who have hyperphosphorylated tau levels similar to carriers of *APOE ε4,4* also have levels of synaptophysin that are similar to those of ε4,4 carriers. The association between high hyperphosphorylated tau, low dynactin-P50, and synaptic loss, as denoted by low synaptophysin levels noted in one of our ε3,3 cases, suggests that factors other than *APOE* genotype are involved in aggregate formation, and may separately contribute to retrograde transport deficits. It is interesting to note that elevation of IL-1β, the bellwether of Alzheimer's neuroinflammation, increases synthesis of MAPKp38 and production of its active phosphorylated form, viz., the form of MAPKp38 that we found necessary for production of hyperphosphorylated tau and for the reduction of synthesis of synaptophysin (Li et al., 2003). In view of this, in the many conditions in which IL-1β levels are elevated, it is logical to predict a corresponding increase in production of hyperphosphorylated tau and a decrease in production of synaptophysin, regardless of *APOE* genotype.

## Conclusion

Age imposes increasing amounts of neural stress, even in a person free of dementia and of neuropathological change. This stress is mitigated in neurons by increasing the levels of the neuronal stress response protein βAPP and its retrograde transport protein dynactin-P50, allowing for retrograde transport of βAPP, which after use, may need to be re-cycled or degraded. However, in Alzheimer's, a combination of factors, including *APOE* genotype, disrupts this stress response system beyond its ability to cope, and in this way, initiates and perpetuates a cycle in which Aβ is generated, and tau hyperphosphorylation and aggregation are favored. These proteins may then stick together, and sequester useful proteins such as dynactin-P50 into aggregates, preventing them from performing their function; this deleterious cycle leads to synaptic loss, and eventually cell death. In addition to the risk factors we mention here, namely, *APOE* genotype, other reports imply other factors can come into play, specifically endosome/lysosome dysfunction (Kimura et al., 2009; Lee et al., 2011), microtubule density reduction (Cash et al., 2003), and decreased dynein/dynactin interaction (Kimura et al., 2007), leading to decreased transport efficiency. This idea points to the possibility of targeting the protein transport system in development of Alzheimer treatments, in addition to its important regulators: inflammation, proteostasis, and stress-response.

Our findings suggest that neurodegenerative events seen in Alzheimer's are related to dysregulation of microtubule-associated motors that carry proteins and other cargo to and from the neuronal soma, causing an accumulation of dynactin-P50, βAPP, and neurofibrillary tangles in the bulbous neurite endings present in the neuritic amyloid plaques diagnostic of Alzheimer's disease. The fact that each of these entities is also present in neuronal somas, especially those adjacent to plaques, suggests that not only retrograde but also anterograde motor systems are impacted in Alzheimer pathogenesis. Our finding that even in disease *APOE ε3,3* plays an ameliorating role in potential for delivery of spent and misfolded proteins may explain, at least in part, the benefits of inheriting this genotype on risk of development of Alzheimer's. Importantly, our findings make clear the possibility of successful aging characterized by necessary changes in transport measures to meet age-related challenges, such as changes in the expression of cargo entities associated with age-appropriate expression of proteins, organelles, and neurotransmitters.

## Author contributions

OA, KM, and PP shared equally in the work and in manuscript preparation, LL performed the westerns and their analyses, RM performed the neuropathological evaluations and contributed to preparation of the manuscript, and WG led the project, data interpretation, and manuscript writing.

### Conflict of interest statement

The authors declare that the research was conducted in the absence of any commercial or financial relationships that could be construed as a potential conflict of interest.

## Footnotes

^1^1997. Consensus recommendations for the postmortem diagnosis of Alzheimer's disease. The National Institute on Aging, and Reagan Institute Working Group on Diagnostic Criteria for the Neuropathological Assessment of Alzheimer's Disease. Neurobiol. Aging 18, S1–S2.

## Support

NIH/NIA AG12411 and the Windgate Foundation.
